# Root causes of deaths by suicide among patients under the care of a mental health trust: thematic analysis

**DOI:** 10.1192/bjb.2020.106

**Published:** 2021-06

**Authors:** Opeyemi Odejimi, Kerry Webb, Dhruba Bagchi, George Tadros

**Affiliations:** 1 Birmingham and Solihull Mental Health Foundation Trust, UK; 2 Aston University, UK

**Keywords:** Root causes, mental health service, suicide prevention, serious incidents, suicide

## Abstract

**Aims and method:**

This study explored the root causes of deaths by suicide among patients under the care of a mental health trust. Thematic analysis was carried out to identify themes from the serious incident reports for patients between 1 January 2017 and 31 July 2018.

**Results:**

In total, 48 cases were reviewed. Three main themes emerged from this study: patient-, professional- and organisation-related factors. The majority of the deaths were caused by patient-related factors, particularly exacerbation of the patient's mental health condition.

**Clinical implications:**

This study provides insight into perceived causes of death by suicide among mental health patients. It is hoped that this will, in turn, influence the manner in which decisions, policies and resource allocation are carried out to further prevent and reduce the incidence of suicide, particularly among mental health patients.

Suicide is a global health problem. It is estimated that every year about 800 000 people die by suicide worldwide.^1^ Previously, the UK has reported a significant reduction in the rate of suicide. However, a report in 2018 indicated a marked increase.^2^ Furthermore, 28% of people who died by suicide in the UK were under the care of a mental health service 12 months prior to their death.^3^ This implies that more than one-quarter of patients who die by suicide have an underlying mental illness and were known to services prior to their death. Perhaps, if timely intervention had been in place, the risk of suicide might have been reduced.

Serious incident reports represent a record of events deemed to have had untoward consequences for patients, families and/or carers, and the organisation.^4^ The reports are produced to identify areas for improvement in order to avoid a recurrence of such events, and not to apportion blame.^4^ Any case of unexplained death is classed as a serious incident and is referred to the coroner for inquest. Following an inquest, the coroner will make a verdict of accident, natural causes, suicide, industrial disease, narrative or open.^5,6^ A suicide verdict is only given by the coroner following evidence from the suicide report indicating that ‘on the balance of probabilities’ the deceased performed and intended an act of suicide that would result in the end of life.^7^ This new standard for the coroner's ruling has been in place since May 2019.^7^

## Predictors of suicide

In most cases, identifying why patients die by suicide is challenging. In fact, the Samaritans^8^ supported this notion, making it clear that the causes of suicide are not straightforward but sometimes could be preventable. In the clinical setting, the SAD PERSONS scale is the risk assessment tool used to predict the possibility of suicide among patients.^9^ However, there remains insufficient evidence of its ability to predict suicide, because it fails to provide a comprehensive understanding of the underlying causes of suicide; however, it continues to be used globally.^10,11^ Thus, an understanding of possible underlying causes of suicide is imperative.

The root cause analysis within a serious incident report provides details about possible or perceived causes of suicide. The root cause is based on systematic investigation of what led to the serious incident. Root causes of suicide can be multifactorial but are generally classed as: individual, quality and safety process, organisational, situational and care-related factors.^12,13^ However, in the light of the complexities associated with suicides, understanding the root causes of suicide may be a step in the right direction to prevent suicide globally.

This study aims to explore common themes emerging from root cause analysis of serious incident reports for mental health patients who died by suicide under the care of a mental health trust. The research question is: what are the root causes of suicide among mental health patients? It is hoped that study will give an indication of the perceived underlying causes of suicides among mental health patients and therefore help service providers, researchers and policy makers to implement policies and strategies to further prevent and reduce the incidence of suicide, particularly among mental health patients.

## Method

**Fig. 1:**
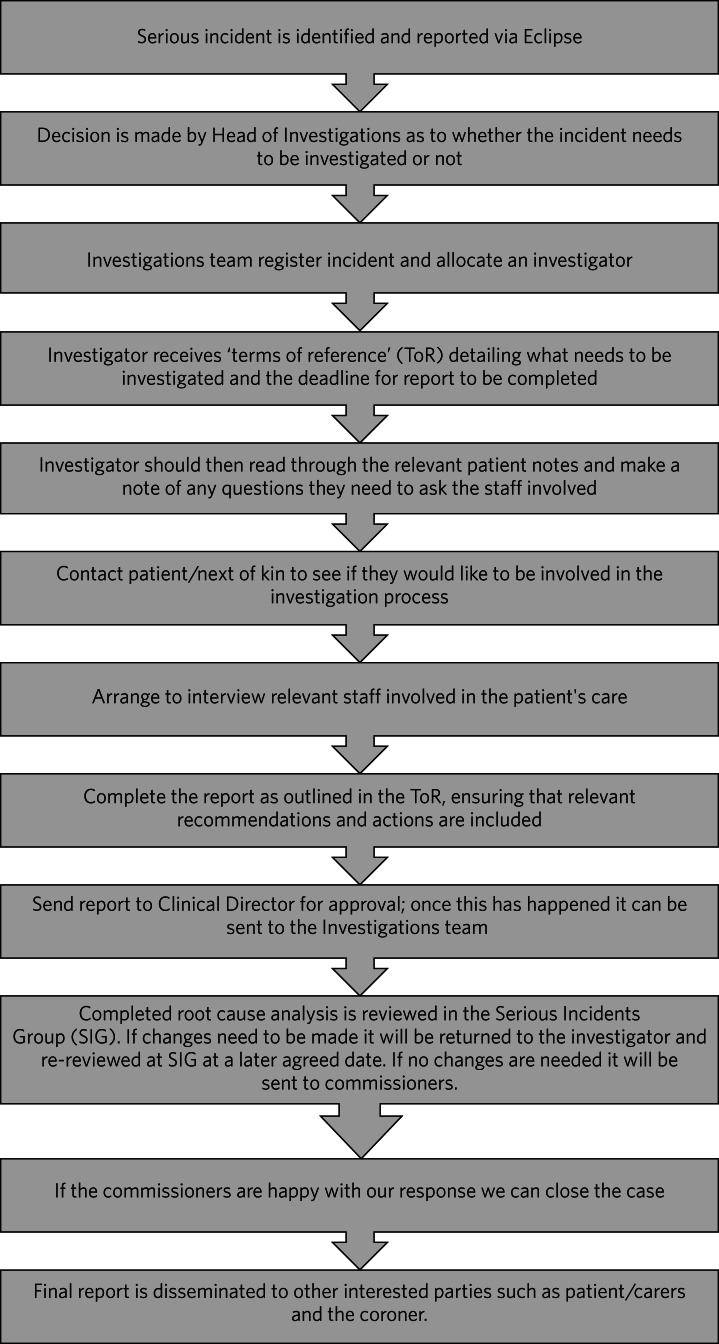
Serious incident report process.^4^

This study took place in a National Health Service trust in the UK's Midlands. Data were collected as part of a service evaluation within the trust. The process of reporting a serious incident within the trust is detailed in Fig. 1. In the trust, serious incident reports are based on evidence gathered independently. Serious incident reports for patients who died by suicide provide details about the individual, the intent, and the event leading up to the suicide, with the hope of learning about the cause(s) of suicide.

Serious incident reports are written by a senior clinician within the trust who was not involved in the care of the patient. The serious incident investigations are done soon after the incident to avoid issues about recollection of events. However, the report is not published until the coroner's verdict has been established. The aim is for a serious incident report to be instigated and completed within 3 months of the event, although the coroner's verdict may take longer to be established. The serious incident report is based on semi-structured interviews with the clinicians, the patient's relatives and loved ones, and examination of the case notes.

The root cause is established by the serious incident review team group, following the investigation by the senior clinician. A typical root cause may be about a paragraph consisting of a few sentences and is very specific about the definitive cause of suicide. The root cause section is not based on a predefined category, and details reported vary among patients. Furthermore, in some cases, a root cause may not be identified, whereas other reports contain more than one root cause. The root causes and other findings such as shortfalls in service and delivery within the serious incident report are later disseminated in team meetings. Actions are then taken based on the recommendations suggested for the learning process and service changes.

In this study, serious incident reports for patients who died by suicide while under the care of the trust between 1 January 2017 and 31 July 2018 were reviewed. There were 71 deaths during this period, of which 36 were ruled as suicide by the coroner. It should be noted that the coroner's rulings of suicide in the serious incident reports reviewed in this study were based on the old standard of ‘beyond all reasonable doubt’. A further 16 serious incident reports with a narrative verdict were considered by the service evaluation team as possible suicides and were therefore included to increase the scope of learning. This review was therefore based on 48 cases.

Thematic analysis was carried out inductively by the authors to identify themes emerging only from the root causes of the serious incident reports.^14^ Thematic analysis was selected owing to its ability to generate trustworthy and insightful rich data about the root causes of suicide among mental health patients.^14^ Moreover, the use of an inductive approach helped to create themes directed by the content and not by preconceived ideas or theory. Braun and Clarke's^14^ six-step procedure was used to identify themes. Familiarisation, coding, theme development, revision, naming and writing up were carried out by the main researcher and agreed by two other authors. Any disagreements were resolved by discussion.

Trustworthiness and rigour were established using Lincoln and Guba's^15^ criteria: credibility, transferability, dependability and confirmability. Credibility was ensured by member checking and triangulation. Member checking was carried out by the review team, who validated the findings of the serious incident reports. Triangulation was ensured by the main researcher discussing the findings with two other authors. If there were disagreements, they were resolved by discussions. Transferability was achieved by providing a detailed description of the research by all authors such that it could be easily applied in other contexts. Dependability was attained by clearly documenting the research process. Confirmability was achieved by ensuring that the interpretations and findings were derived from the data, with themes and subthemes supported by quotes.

### Ethical considerations

This study was scrutinised and approved by the Research and Innovation Department of the trust. Information from serious incident reports was only disclosed to the review team. Data were protected by storing electronic data on an encrypted USB drive and password-locked computers, and paper files were stored in a locked cabinet. All materials relating to this service evaluation will be stored for at least 3 years from the end of the study in accordance with the trust's research policy.

## Results

There were nearly twice as many deaths in males (*n* = 31) as in females (*n* = 17). The age range was 15–86 years. The most common method of suicide was hanging. Two-thirds of the death took place at home (*n* = 32), and only one death occurred in the hospital on an in-patient ward. It should be noted that one-sixth (*n* = 6) of the reports had no identified root cause. Three main themes emerged, each of which had a number of subthemes. Quotes only from the root cause sections of the serious incident reports were used to support the subthemes and themes emerging in this study. Quotes from each serious incident report were assigned a code. The serious incident reports were labelled in chronological order (1–48). The three main themes emerging from this study were: patient-, professional- and organisation-related factors.

### Patient-related factors

The thematic analysis identified three main patient-related factors that contributed to deaths by suicide: exacerbated mental health conditions, lack of engagement with services and non-adherence to medications. An exacerbated mental health crisis was the most common patient-related factor and also the most frequently recurring subtheme emerging from the serious incident reports. The exacerbated mental health condition was often secondary to physical health problems, social and relationship difficulties, an underlying criminal offence, alcohol and substance misuse, or sexual offences, especially child pornography.

Furthermore, in some cases where lack of engagement and non-adherence to medication were identified as root causes, patients also had an exacerbated mental health condition. This is because these factors could have a bi-directional effect. For instance, lack of engagement and non-adherence to medication could result in exacerbated mental illness and *vice versa*.

‘*Patient had a history of being reluctant to come into hospital. Deterioration in mental health was triggered in response to an argument with his family; the patient had an argument with a family member, several days prior to his death’ – Report 24*

‘*The patient suffered from paranoid schizophrenia, discontinued depot medication and thereafter appears to have complied poorly with oral medication. There was a two month period during which no medication were taken’ – Report 16*

### Professional-related factors

These are factors related to the manner in which care and services are delivered by mental health professionals (usually doctors and nurses). Five main factors emerged: issues around risk assessment and management, inadequate clinical enquiries, non-adherence or poor adherence to policies and procedures, no interprofessional communication and collaboration, and lack of consultation of carers by clinicians.

Among these five factors, issues around patient assessment and management were the most common and represented the second most frequently recurring subtheme in this study. This was an interesting finding, as most mental health professionals are trained and are aware of the protocols and practices within the trust. Further exploration revealed that poor practice, especially improper documentation, was a major cause. This was noticed more in cases where the patient frequently presented and the health professional did not update the risk assessment and management documentation.

‘*Early warning signs were not documented, so it is unclear whether these were identified, known and shared. Clinical documentation provided wrong information (details were not updated) about patient current state. It was anticipated that patient would make a full recovery having presented frequently previously’ – Report 21*

Further exploration into cases where professionals did not carry out enough detailed clinical enquiries shows that clinicians had misperceptions regarding the seriousness of the patient's presentation. This clinical enquiry includes patient history and collateral information from carers and other professionals managing the patient.

‘*The patient superficially appeared to be making some progress and was fully compliant with care plan and activity schedule and was engaging well with staff and patients. There was no indication that progress would not continue. It was anticipated that the patient would make a full recovery’ – Report 15*

Consulting carers may help clinicians to corroborate or contradict patients’ claims about their mental health. Carers may be family, friends or any loved ones that look after patients in an non-professional capacity. In this study, one report indicated that the root cause was the clinician not carrying out detailed clinical enquiries and not consulting the patient's carer.

‘*Patient concealed information and gave assurance of not been suicidal. This was taken on face values, despite family expressing concerns and partners assertion that patient was concealing information’ – Report 43*

Clinicians mostly did not adhere to the trust's follow-up review standards. Follow-up is very important as it ensures that a patient receives continued support whether they are in crisis or not.

‘*Patient was discharged from mental health treatment with no follow-up or aftercare arrangements despite agreement to liaise with Hospital X, and despite further episode of self-harm and having been pending transfer from hospital Y to a mental health bed’ – Report 11*

Likewise, a lack of interprofessional communication and collaboration may result in the patient not having the necessary support. This is particularly important for patients who have comorbid physical and mental health problems.

‘*Given the patient consistently gave differing accounts of history to a multitude of practitioners, the trust should have engaged with other agencies involved in patient care and this will have helped deliver holistic care’ – Report 20*

### Organisation-related factors

Factors within the organisation identified as root causes included inadequate psychiatric accommodation, a lack of additional support for mental health patients (such as drug and alcohol services) and unsafe wards. At the time this study was conducted, the trust did not have a drug and alcohol service, although patients were often referred to local available services. Nevertheless, patients and their carers often indicated that they were not satisfied with the referred drug and alcohol services and would prefer one within the trust.

‘*Patient indicated not finding the drugs and alcohol services helpful but was still signposted. It is highly unlikely patient will make self-referral and engage with the services’ – Report 9*

Psychiatric ward bed shortages in the trust can be attributed to high demand, which may be a result of the trust's location in one of the main cities within the West Midlands. It should also be noted that in some cases where psychiatric beds were not immediately available, alternatives such as daily visits within the community or a short stay in a general hospital ward or mental health supported accommodation in the community were provided to patients. Providing such alternatives may be a safe alternative in the absence of the ideal psychiatric ward admission; however, the care and support provided in such an environment may not be suited to the patient's needs. This does not exclude the fact that suicide occurs in in-patient wards.

‘*The patient had a complex history and had taken overdoses previously, The last contact with the Trust, patient was expressing concerns about sleep again and was on the bed list and daily review with HTT but perhaps would have benefited more as inpatient on the ward’ – Report 36*

In this study, there were two cases of unsafe wards, one an in-patient ward within the trust and the other in prison accommodation. It was further observed that the in-patient death occurred as a result of not adhering to the trust's observation policy.

‘*Patient had serious mental illness, personality disorder and substance use disorder, There was no observation in last 24 hour before death and no evidence of psychiatric and psychological input’ – Report 41*

Thus, in-patient wards may be a safer option for reducing deaths by suicide; however, if no other patient- and professional-related factors are taken into consideration, in-patient wards may not provide the necessary prevention of suicide among mental health patients.

## Discussion

This study confirms views about suicide being a complex problem, with aetiology and predictors that are difficult to identify.^13,16,17^ The root causes considered in this study will provide a more comprehensive understanding of possible underlying causes of suicide than the SAD PERSONS scale. This is because the SAD PERSONS scale appears to focus more on certain patient-related factors,^9,10^ whereas this study identified that underlying professional- and organisation-related factors can also influence suicide rates.

The findings are consistent with those of previous studies regarding acute crisis having a strong association with suicide among mental health patients.^13,18^ Thus, there is a need for continued assessment and support in mental health services. Often, a patient who died by suicide deliberately did not give essential information or denied plans for suicide when assessed by health professionals.^16,19^ This can be particularly difficult, as those patients are very likely to be frequent attendees with a history of self-harm, suicide ideation and multiple suicide attempts. Meanwhile, health professionals want to respect and listen to patients; hence, they work with the details provided by the patient, which might not give a true picture of the extent of their symptoms.

A particularly significant finding of this study is the need for processes, procedures and training that help health professionals to increase their ability to carry out detailed clinical enquiries while assessing and managing patients. Also emerging from this study is the need for an approach to patient and carer involvement that promotes active participation of patients and their carers (family, friends or loved ones) in assessment and management of patients. The National Confidential Inquiry into Suicide and safety in Mental Health (NCISH)^3^ report also supports this view, stating that clinicians should conduct a robust patient assessment which is person centred and takes into consideration the stressors, support and perspectives of family and carers.

Furthermore, involving family members is particularly useful in corroborating or contradicting the symptoms expressed by patients,^20^ especially when patients do not willingly divulge information or deny the extent of their mental health crisis. However, confidentiality and carer rights are two factors that can swerve professionals in their decision about the extent to which family members should be involved. Also, clinicians should explore whether family member involvement in patient assessment and management is a potential protective or risk factor.^16^

Shortage of beds was not a major reason for deaths by suicide in this study. Since the closure of asylums in the UK in the 1950s, more mental service provision now occurs in the community than on in-patient psychiatric wards.^21^ Thus, it can be presumed that community and outreach services are equally effective in managing mental health patients and reducing avoidable admissions. It can be argued that patients in crisis will receive more effective care and support in a psychiatric unit than at home.^22^ Nevertheless, each patient should be evaluated based on their risk and triggers, and a decision should be made regarding whether intervention services should be delivered in the community or in a psychiatric unit.

Adherence by health professionals to policies and procedures is a crucial aspect of reducing deaths by suicide. For example, where handover and referral procedures are not adhered to, insufficient details will be passed on, affecting interprofessional communication and collaboration. Ultimately, this may result in patient assessment not being holistic, with a spiral effect on the management of the patient. Perhaps the reason the discharge and handover policies were not adhered to was poor clinical documentation. Fowler^16^ emphasises the importance of proper clinical documentation in providing comprehensive and practical patient assessment and management. Thus, this study indicates that clinical documentation can have a spiral effect on the assessment and continuity of care of patients and on suicide prevention.

The NCISH^3^ report recommends safe wards and early follow-up as key ways of reducing suicide incidence. Considering that hanging is the most common method of suicide in the UK, having safer wards is an essential priority to reduce incidence on the wards. This view is also consistent with the study of Meehan *et al*,^23^ who suggested that in-patient wards should be redesigned to ensure safety. However, it appears that more suicides take place at home than elsewhere, as revealed in this study. Although it might not be practical to design or redesign all mental health patients’ homes to be safe, other measures need to be put in place. For instance, studies have shown that there is higher risk of suicide in the first 7 days after discharge.^3,23,24^ Thus, carrying out early follow-up should become a priority in suicide prevention.

### Summary

Suicide prevention remains a priority globally. Investigating root causes is a step in the right direction in developing strategies that may be effective in reducing the current suicide rate. It is acknowledged that root causes are not conclusive evidence of the reason suicide occurs; nevertheless, they provide an indication of the underlying causes of suicide.

The three major root causes identified in this study are interwoven, and the goal should be for suicide prevention strategies to take into cognisance all three factors. However, it is also recognised that in the present economic situation, resources are scare. This study adds new knowledge about suicide prevention by highlighting root causes of suicide among mental health patients. It provides insight into the two most likely root causes, which are exacerbated mental health conditions and issues around patient assessment and management.

Moreover, this study indicates that using a robust person-centred approach with involvement of carers (family, friends or loved ones) in assessment and management, especially among frequent attendees, may help to prevent suicide in mental health patients. Furthermore, this study highlights the need to carry out a risk assessment each time a patient presents, in order to have an updated and relevant patient safety plan. Even in scenarios where patients present on several occasions and no new risks or triggers are identified, health professionals should document this, and a rationale for not giving an update should be provided in the patient record.

A limitation of this study was that it was conducted in one hospital trust in the West Midlands region of the UK. Therefore, the findings may not be generalisable to all other mental health services. Nevertheless, the findings are transferrable and could be applicable to other mental health services. Another limitation was the flexibility of the thematic analysis, which allows researchers to use what is deemed applicable to their research aims and objectives. To minimise this limitation, the research team have provided justifications for the choice of this methodology and details of the data analysis, and explained the measures taken to ensure trustworthiness and rigour.

A suggestion for further research is to identify factors that make patients more vulnerable to suicide in non-hospital settings and provide evidence-based strategies to reduce these. Overall, this study provides insight into perceived causes of death by suicide among mental health patients. It is hoped that this will in turn influence the manner in which service providers, researchers and policy makers carry out decisions, policies and resource allocation and implement strategies to further prevent and reduce the incidence of suicide, particularly among mental health patients.

## Data Availability

All data generated or analysed during this study are available on request.

## About the authors

**Opeyemi Odejimi** is a researcher in the Psychiatric Liaison Department, Birmingham and Solihull Mental Health Foundation Trust, UK; **Kerry Webb** is Suicide Prevention Lead in Acute Care Mental Health, Birmingham and Solihull Mental Health Foundation Trust, UK; **Dhruba Bagchi** is Clinical Director at the Birmingham and Solihull Mental Health Foundation Trust, UK; and **George Tadros** is Professor of Psychiatry and Dementia at Aston Medical School, Aston University, UK.
